# Contrast‐Enhanced Ultrasound Predicts Surgical Margin Positivity in Patients With Breast Cancer Who Underwent Partial Mastectomy

**DOI:** 10.1002/wjs.12628

**Published:** 2025-05-19

**Authors:** Hiroaki Shima, Fukino Satomi, Daisuke Kyuno, Noriko Nishikawa, Satoko Uno, Yuta Kondo, Ai Noda, Takashi Nakamura, Toru Mizuguchi

**Affiliations:** ^1^ Depertment of Surgery, Surgical Oncology and Science Sapporo Medical University Sapporo Japan; ^2^ Department of Pathology Sapporo Medical University Sapporo Japan; ^3^ Sapporo Kitaguchi Clinic Sapporo Japan; ^4^ Muroran City General Hospital Muroran Japan; ^5^ Otaru Ekisaikai Hospital Otaru Japan; ^6^ Deptertment of Nursing, Surgical Science and Technology Sapporo Medical University Sapporo Japan

**Keywords:** breast cancer, contrast‐enhanced ultrasound, partial mastectomy, surgical margin

## Abstract

**Background:**

The clinical disadvantage of positive margins in partial mastectomy for patients with operable breast cancer is clear and must be avoided; however, there is still room for improvement. The usefulness of contrast‐enhanced ultrasound (CEUS) in diagnosing spread is currently well‐known. The CEUS‐enhanced area for breast cancer tends to be wider than that observed in B‐mode US and probably includes cancer cells. Therefore, we focused on the difference obtained by subtracting the maximum diameter on B‐mode US from that on CEUS. This parameter tends to be greater than zero. However, there are tricky cases in which such enhancements are not visible, and the enhanced area remains limited to a small region. This study aimed to analyze the correlation between characteristic findings and positive for margins in order to ultimately prove potential usefulness of CEUS in making the surgical margin negative.

**Methods:**

We retrospectively evaluated the data of consecutive 142 patients with breast cancer who underwent partial mastectomy to explore the effect on positive margins when the CEUS enhancing area was smaller than the B‐mode US visualized mass (CEUS‐B ≤ 0).

**Results:**

Positive surgical margins were observed in 14 out of 142 patients. CEUS‐B ≤ 0 was associated with significantly more positive margins (*p* = 0.0467). CEUS‐B was also extracted as an independent predictor on multivariate analysis.

**Conclusions:**

The findings of no enhancement outside the area of visible tumor on CEUS but not visualized outside the area of visible tumor on B‐mode US might be a risk factor for a positive surgical margin.

## Graphical abstract

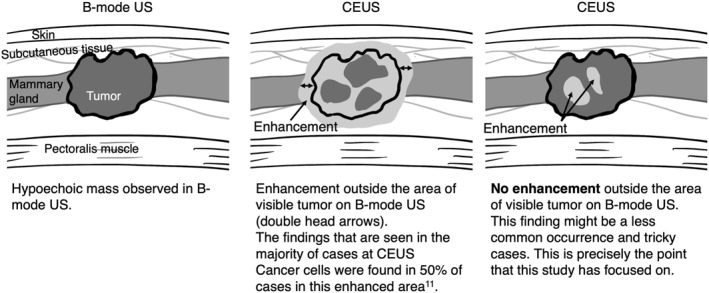

## 1 Introduction

Partial mastectomy is a standard procedure in breast cancer surgery; however, studies have demonstrated that the local recurrence rate is approximately twice as high in patients with cancer positive for margins as that in patients with negative margins [1, 2]. Therefore, the guidelines recommend ensuring negative margins [3, 4]. Another meta‐analysis suggested that surgeons should aim to achieve a clear margin of at least 1 mm of invasive breast cancer [5]. Hence, margin clearance remains a definitely important issue for breast surgeons. The current standard diagnosis for the extent of disease is a comprehensive diagnosis based on imaging obtained from magnetic resonance imaging (MRI), mammography, and ultrasonography (US); however, unfortunately, the rate of positive margins has not reached zero. Several new complementary imaging techniques have been reported as useful modalities in the preoperative spread diagnosis of breast cancer, such as positron emission mammography and contrast‐enhanced spectral mammography [6, 7]. Positron emission mammography has been reported to have almost a similar diagnostic performance as that of MRI, which is inferior to that of MRI in terms of sensitivity but superior in terms of specificity [8]. The advantage of contrast‐enhanced spectral mammography is that its detection ability is comparable to that of MRI, and it is reported to exhibit higher specificity and fewer false positive results than MRI [9]. Because both modalities require imaging in a different position from the surgical position, there are still some issues to be addressed. Despite the advantages and disadvantages of each modality, B‐mode US is a simple examination that can be performed while the patient is in the surgical position and which also provides high resolution, so it can be easily used in daily clinical practice. However, the problem of negative margins has still not been entirely solved as mentioned above. However, contrast‐enhanced ultrasound (CEUS) makes it possible to see the blood flow in tiny blood vessels [10]; therefore, we think it is a promising modality among those that are available for practical implementation.

**FIGURE 1:**
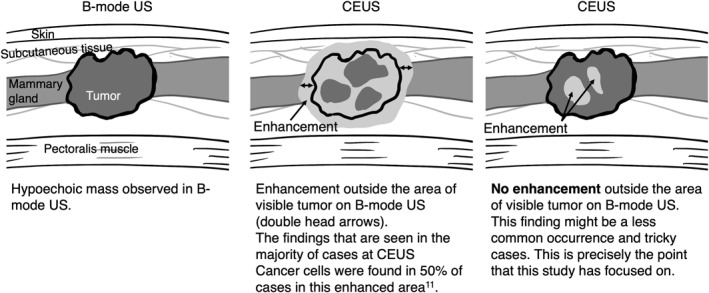
Characteristics of CEUS compared to B‐mode US. When observed with CEUS, it is generally indicated with “enhancement outside the area of visible tumor on CEUS but not visualized outside the area of visible tumor on B‐mode US”. On the other hand, no enhancement outside the area of visible tumor on B‐mode US might be a less common occurrence and tricky case. This finding is the focus of this study.

A study reported that CEUS is potentially useful in the diagnosis of spread, as the enhanced area of CEUS tends to be wider than that of the usual B‐mode US and closer to the maximum diameter for pathological diagnosis [11]. In the same study, as an exploratory analysis, pathologically cancer cells have been detected in approximately half of the patients within the area showing contrast effects on CEUS [11]. It can be thought that CEUS more accurately visualizes the extent of breast cancer pathologically than B‐mode US, as it adds blood flow information to the area observed in B‐mode. Therefore, it might be considered reasonable to include the range of surgically resected specimens. Nevertheless, there are some cases in which the range of enhancement is not wider than the area of visible tumor on B‐mode US, and it is not clear how this should be interpreted Figure 1. In this study, we defined a parameter “CEUS‐B,” which represents the maximum diameter enhanced on CEUS minus the maximum diameter visualized on B‐mode US, and used this parameter for analysis.

Because MRI allows the localization of lesions of the entire breast, mapping and resection area settings are often based on this image. Nevertheless, because MRI is often performed in a different body position from the surgical position, misalignment between the images and findings has been a concern in clinical practice [12, 13, 14, 15, 16]. This study was designed to explore what aspects of preoperative diagnosis of cancer spread to determine the extent of resection in the breast might be useful in reducing the number of positive margins in partial mastectomy. We focused on patients in whom CEUS demonstrate an unusual enhancement when used as additional information to MRI.

## 2 Patients and Methods

### 2.1 Procedures

This was a single‐center, retrospective observational study conducted to investigate the surgical margin positive rate for patients with breast cancer, who underwent partial mastectomy, using CEUS in addition to mammography, B‐mode US, and MRI. Key inclusion criteria were breast cancer diagnosed pathologically, patients who underwent partial mastectomy, and patients who underwent examination for the extent of the cancerous lesion to be added to the resection area using CEUS and B‐mode US. Typically, the patient was positioned on the operating table rotated to elevate the affected side and the head erect position as the surgical position under anesthesia in the operating room. Patients were excluded if they met any of the following criteria: patients undergoing any preoperative systemic therapy, patients undergoing preoperative irradiation, and patients who had requested not to participate in this study by written regretted opt‐out.

B‐mode US and MRI were performed to evaluate the extent of the cancerous lesion before surgery. To evaluate the primary breast tumor, MRI was performed with frequency‐selective fat suppression (e‐THRIVE) and maximum intensity projection imaging, and the contrast effect range was judged as a viable lesion (Ingenia 3.0 T Ω HP release 5.41, Philips Healthcare). B‐mode US was performed by laboratory technicians who reported the imaging data in clinical documents using Aplio 700 and Aplio a550 (Canon), and CEUS was performed by the surgeons using LOGIQ E9 (GE Healthcare Japan).

In the operating room, a comprehensive evaluation was conducted with reference to the B‐mode US and MRI already taken, and then the CEUS and B‐mode US was performed in the surgical position to confirm the details of the spread. As CEUS requires intravenous infusion of a contrast agent, it is performed in the operating room after securing vascular access during anesthesia induction. This allows the examiner, who is also the surgeon performing the operation, to directly observe the findings in real time. When determining the resection area, we specifically consider the enhanced region on CEUS as the estimated tumor and secure a 1.5‐cm margin from the enhanced area for excision, based on preoperative all image findings.

The technique for partial mastectomy involves a resection of a 1.5‐cm safety margin in all lateral directions centered on the area to be enhanced by CEUS, with reference to the MRI and US already performed. Furthermore, on the chest wall side, the pectoralis major fascia included a layer for resection, whereas the pectoralis major muscle was preserved. Xeroradiography was performed in a routine manner on the excised specimens to confirm that they had been appropriately resected, and the intraoperative histological examination of the three transected margins was also performed to ensure that they were appropriately resected.

Pathological findings included the diameter of the remnant lesions in the surgical pathology report prepared by pathologists. The excised specimens were sectioned in the nipple–tumor orthogonal direction into 5‐ to 7‐mm sections. The extent of the lesions on the sections was evaluated by pathologists. The specimens were photographed side by side in sequence and mapped by a pathologist based on the corresponding pathological spread of the cancer. Positive margins were defined as the proximity of the in situ lesion to the margins within 2 mm or exposure of invasive cancer [3, 4]. We analyzed how the relationship between the maximum transverse diameter of the CEUS‐enhanced area and the maximum transverse diameter of the B‐mode US is involved in causing positive margins.

The study protocol was approved by the Clinical Trial Center of Sapporo Medical University, Japan, and was conducted according to the Declaration of Helsinki and the Ethical Principles for Medical Research Involving Human Subjects (Ministry of Health, Labor and Welfare). This study was also approved by the Clinical Research Review Committee of the hospital and by the hospital director and is registered with UMIN‐CTR (UMIN000053287). In this study, as it focuses on breast cancer patients, male breast cancer is extremely rare. Therefore, all eligible cases are female. This study complies with the guidelines of the Surgery Journal Editors Group.

### 2.2 Statistical Analysis

To explore whether the CEUS‐enhanced area was smaller or larger than visible on B‐mode US might be related to the surgical positive rate, a χ^2^ square test was performed for the surgical positive rate in patients with and without CEUS‐B ≤ 0. A χ^2^ test was also conducted for MRI‐B ≤ 0 in the same manner. Two‐sided *p* < 0.05 was considered statistically significant. Odds ratios and 95% confidence intervals for surgical margin positivity for each clinicopathological factor, including CEUS‐B ≤ 0 and MRI‐B ≤ 0, were calculated by nominal logistic analysis. All statistical analyses were conducted using JMP 16.0 (SAS Institute Inc., Cary NC, USA). The receiver operating characteristic (ROC) curve, which indicates the accuracy of the CEUS diagnosis for a positive margin, was obtained by varying the cutoff according to the US findings.

## 3 Results

### 3.1 Study Design

**FIGURE 2:**
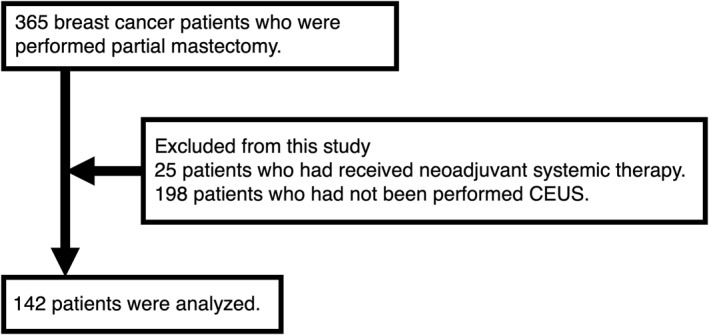
Flow chart of the study design.

Patients with breast cancer who underwent partial mastectomy were enrolled in this study from October 2014 to July 2021. A final sample of 142 patients was analyzed, who had been evaluated by CEUS in operating rooms, who had not undergone previous systemic therapy, and who had not been examined by CEUS (Figure 2).

### 3.2 Patient Characteristics

Table 1 lists the patients' characteristics. The mean age was 55 years. The maximum preoperative tumor diameter on B‐mode US was 29 mm. The largest number of diagnoses histologically was invasive ductal carcinoma (*n* = 124) followed by ductal carcinoma in situ (*n* = 9). Regarding the special type, patients were diagnosed with four invasive lobular carcinomas, two mucinous carcinomas, two invasive micropapillary carcinomas, and one apocrine carcinoma. Excluding the nine ductal carcinoma in situ cases, the most common subtype was ER+/HER2− with 101 cases (71.1%).

**TABLE 1:** Patients' characteristics.

*n* = 142		*n*/mean ± SD	%	CEUS‐B ≤ 0 mm	*n* = 66	CEUS‐B > 0 mm	*n* = 76	
Age	y.o.	55 ± 11		54 ± 11		56 ± 11		*p* = 0.4763
pT	0	9	6.3%	2	3.0%	5	6.6%	*p* = 0.5792
	1	108	76.1%	53	80.3%	57	75.0%	
	2	25	17.6%	11	15.7%	14	18.4%	
pN	0	129	90.8%	61	92.4%	68	89.5%	*p* = 0.5431
	1 ≤	13	9.2%	5	7.6%	8	10.5%	
Nuclear grade	1–2	121	85.2%	54	81.8%	67	88.2%	*p* = 0.2885
	3	21	14.8%	12	18.2%	9	11.8%	
Lymphovascular invasion	0	124	89.2%	56	86.2%	68	91.9%	*p* = 0.2766
	1	15	10.8%	9	13.8%	6	8.1%	
	Missing data	3		1		2		
Ki67	%	19.3 ± 19.0		20.1 ± 20.4		17.9 ± 17.7		*p* = 0.3350
Histology	DCIS	9	6.3%	3	4.6%	6	7.9%	*p* = 0.6018
	IDC	124	87.3%	59	89.4%	65	85.5%	
	Invasive lobular carcinoma	4	2.8%	2	4.6%	2	2.6%	
	Mucinous carcinoma	2	1.4%	2	1.4%	0	0.0%	
	Invasive micropapillary carcinoma	2	1.4%	0	0.0%	2	2.6%	
	Apocrine carcinoma	1	0.7%	0	0.0%	1	1.3%	

Invasive disease excluding 9 DCIS patients								
*n* = 133		*n*/mean ± SD	%	CEUS‐B ≤ 0 mm	*n* = 63	CEUS‐B > 0 mm	*n* = 70	
ER	Positive	118	88.7%	53	84.1%	65	92.9%	*p* = 0.1101
	Negative	15	11.3%	10	15.9%	5	7.1%	
PgR	Positive	104	78.2%	48	76.2%	56	80.0%	*p* = 0.9592
	Negative	29	21.8%	15	23.8%	14	20.0%	
HER2	Positive	22	16.5%	8	12.7%	14	20.0%	*p* = 0.2578
	Negative	111	83.5%	55	87.3%	56	80.0%	
Subtype	ER+/HER2−	101	75.9%	48	76.2%	53	75.7%	*p* = 0.2058
	ER+/HER2+	17	12.8%	5	7.9%	12	17.1%	
	ER−/HER2−	10	7.5%	7	11.1%	3	4.3%	
	ER−/HER2+	5	3.8%	3	4.8%	2	2.9%	

### 3.3 Frequency of Positive Margins and Details

**FIGURE 3:**
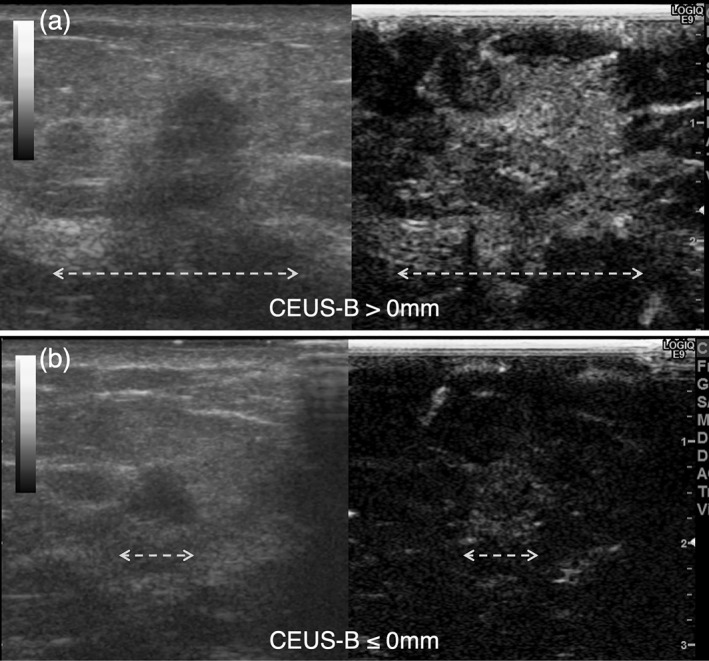
(a) A case with almost no CEUS enhancement; (b) a case with CEUS enhancement extending outside the tumor periphery; in this case, the CEUS enhancement lesion measured 14 mm longer than in B‐mode US. The dashed line is the maximum diameter of CEUS and the dashed line is the maximum diameter of B‐mode US.

Positive surgical margins were observed in 14 patients, whereas negative margins were observed in 128 patients. Of the 14 positive cases, 8 were within 2 mm of the surgical margin, and all these 8 were in situ lesions. In contrast, six cases were exposed, five were in situ lesions, and one was lobular carcinoma in situ, as demonstrated in Table 2. Typical images are shown for cases with wide and not wide CEUS enhancement in Figure 3.

**TABLE 2:** Pathological margin status of the tumor.

	2 mm from ink	On ink
Invasive lesion	0	0
In situ lesion	8	5
LCIS	0	1
Total	8	6

**FIGURE 4:**
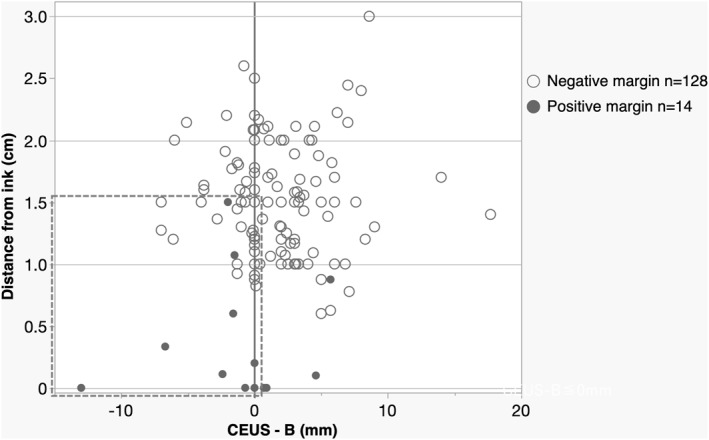
CEUS‐B, that is, the maximum diameter of the enhanced area outside the tumor is placed on the *x*‐axis, and the distance from the tumor to the lateral margin (0 indicates cancer exposure and positive for margin) is placed on the *y*‐axis. Black dots indicate cases with positive margins (including positive cutaneous margins other than lateral margins), and small gray rings indicate those with negative margins. The dotted squares indicate that there are several positive margins in the area surrounded by the dotted squares.

Figure 4 illustrates a plot of subtracting the maximum diameter of the B‐mode US from the maximum diameter of the CEUS (the range of enhancement outside the area of visible tumor on CEUS but was not visualized outside the area of visible tumor on B‐mode US Figure 1; the *x*‐axis) and the distance to the lateral transection (the *y*‐axis). The black circles indicate cases positive for the surgical margin, which tended to be distributed in the range where CEUS‐B is < 0. CEUS‐B was ≤ 0 in 66 of 142 patients; of these 66 patients, 10 showed positive margins, whereas the remaining 76 showed CEUS‐B > 0, and 4 showed positive margins (*p* = 0.0467). In contrast, 55 of 142 patients had an MRI‐B reading of ≤ 0. Of these 55 patients, 4 had positive margins, whereas the remaining 87 showed MRI‐B > 0, and 10 showed positive margins (*p* = 0.4024).

### 3.4 Factors Associated With Risk for Positive Disconnections

Table 3 shows the results of univariate and multivariate analyses for positive margins. In the univariate analysis, the only significant risk factor for surgical margin positivity was CEUS‐B ≤ 0, but not MRI‐B and other clinicopathological factors. In terms of B‐mode‐based imaging diagnosis of tumor extension in operating rooms, a multivariate analysis was performed to compare CEUS and MRI for surgical margin positivity, which indicated CEUS as an independent factor.

**TABLE 3:** Univariate and multivariate analyses of positive margin status.

	Univariate analysis					Maltivariate analysis		
		Vs	OR	95%CI	*p* value	OR	95%CI	*p* value
Age	50 ≤	50 >	3.19	0.50–62.03	*p* = 0.2430			
pT	pTis	pT1 ≤	2.88	0.40–13.63	*p* = 0.2555			
pN	pN1 ≤	pN0	1.34	0.23–25.47	*p* = 0.7765			
ER	Positive	Negative	0.47	0.13–2.28	*p* = 0.3193			
PgR	Positive	Negative	0.48	0.15–1.67	*p* = 0.2363			
HER2	Positive	Negative	0.8	0.12–3.29	*p* = 0.7775			
Nuclear grade	3	1–2	1.67	0.35–5.98	*p* = 0.4918			
Lymphovascular invasion	1	0	0.61	0.03–3.44	*p* = 0.6254			
Histology	DCIS	IDC	3.26	0.44–15.82	*p* = 0.2125			
	Special	IDC	3.26	0.44–15.82	*p* = 0.2125			
CEUS‐B	< 0 mm	0 mm ≤	3.74	1.19–11.84	***p* = 0.0250**	3.91	0.07–0.84	***p* = 0.0239**
MRI‐B	< 0 mm	0 mm ≤	0.6	0.16–1.91	*p* = 0.4024	0.43	0.11–1.43	*p* = 0.1746

*Note:* The bolded parts indicate *p* < 0.05.

### 3.5 CEUS‐B Is a Possible Predictor of Positive Surgical Margins

**FIGURE 5:**
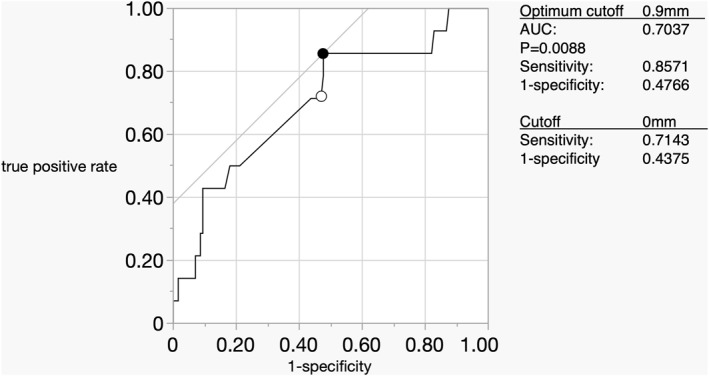
ROC curves calculated using the sensitivity and 1‐specificity of CEUS‐B for a positive cutoff are shown. When the cutoff is set to 0, that is, when the contrast area does not extend beyond the mass visible in B‐mode US, it is a possible predictor of positive margins in the form of AUC = 0.7037, sensitivity = 0.8571, 1‐specificity 0 = 0.4766, and *p* = 0.0088.

The ROC curve revealed that a 0.9‐mm CEUS‐B cutoff of CEUS best served as a surrogate for positive surgical margins, with the area under the curve being 0.7037. Because 0.9 mm may not be clinically meaningful, a sensitivity of 0.7143 and a specificity of 0.4375 were obtained when 0 mm was used as the cutoff (Figure 5).

## 4 Discussion

This study was conducted to explore the usefulness of CEUS in the preoperative diagnosis of breast cancer spread to reduce the number of positive margins in partial mastectomy. In particular, our focus was on unusual enhancement patterns in CEUS. We found a characteristic tendency for patients who did not demonstrate enhancement.

When the CEUS‐enhanced area was smaller than the range visible on B‐mode US (CEUS‐B ≤ 0), it was the only statistically significant risk factor for positive margins in the univariate analysis. This is also a relatively good area under the curve even for ROC curves, and it might be a predictor of a positive margin. In contrast, when the enhanced area as identified by MRI was smaller than that on the B‐mode US, it was not a risk factor. It was also extracted as a statistically significant risk factor for positive margins in the multivariate analysis of both.

As for margin clearance, we used mammography (supplementary), MRI, B‐mode US and CEUS to comprehensively diagnose the extent of the cancer focus, and as a matter of principle, the tumor was resected with a safety margin of 1.5 cm from the estimated tumor margin. This is a widely accepted method in Japan. The immediately preoperative use of B‐mode US and CEUS helps minimize positional discrepancies. Furthermore, it allows for the immediate resolution of examiner‐dependent issues such as the lack of information regarding images that other examiners had not applied but which the operator requires, and this of course represents a weakness of ultrasound testing itself.

Since excessive resection can reduce the esthetic appearance of the breast, it is important to determine the extent of resection in a partial mastectomy without over‐ or under‐resection. There were 14 cases with positive margins, with the exception of one case of LCIS and the other 13 cases were in situ lesions. The most likely fact might be that these cases were diagnosed as having positive margins because the extent of in situ spread could not be correctly diagnosed using these modalities. Conversely, the invasive lesions had been successfully resected and the margins were clear. It is known that low‐grade in situ lesions, and in some cases, invasive lesions, may not be enhanced on MRI. A randomized controlled trial comparing preoperative MRI assessment with nonMRI evaluation was conducted; however, it did not demonstrate a statistically significant reduction in re‐intervention rates for the primary endpoint [12]. Notably, 82% of the cases involved nonmass lesions, making accurate diagnosis by MRI itself challenging. Therefore, the diagnosis of in situ lesions remains difficult. CEUS is considered a promising modality that could contribute to improving the accuracy of comprehensive diagnosis combined with mammography, B‐mode US and MRI.

As for the differences between MRI and CEUS, one feature is the difference in contrast agents. CEUS is a modality that can evaluate lesions and the surrounding hemodynamics in real time [10]. Sonazoid is a second‐generation US contrast agent with excellent in vivo stability and resistance to US sound pressure. It is composed primarily of microbubbles and perflubutane gas encapsulated in a film of sodium hydrogenated egg yolk phosphatidylserine [10, 17], with an average particle size of 3–5 μm, and unlike the water‐soluble contrast agents used in breast MRI, Sonazoid does not leak out of the blood vessel [10, 11, 17]. In other words, it is reported that CEUS can detect tumor‐associated angiogenesis in smaller blood vessels. In many cases, the contrast effect of CEUS extends to the area around the tumor [10]. In situations where this is not the case, there is a possibility that there are micro vessels that are not contrasted by either MRI or CEUS, and we hypothesized that in irregular cases such as this CEUS‐B < 0, there were many instances of in situ lesions with positive margins [18, 19]. It has been reported that MRI has high sensitivity but low specificity, and this is because the contrast agent used in MRI can leak outside the blood vessels [20]. On the other hand, the advantage of microbubbles in CEUS is that they do not leak outside the blood vessels. Therefore, the areas that are enhanced in CEUS may be more accurate than those in MRI, and the fact that it can be performed in the surgical position is a major advantage. The fact that the margins were not positive in the invasive lesions encountered in this study using CEUS is also significant. Regarding the extent of resection within the CEUS‐enhanced area, CEUS‐B ≥ 0 cases are important to ensure that the enhanced area is included in the resection area. However, CEUS‐B < 0 cases, which are the focus of this study, include tricky cases with undetected widths in situ lesions. It is necessary to perform comprehensive diagnosis using mammography, US and MRI, and avoid underestimating the in situ lesion. A heterogeneous enhanced effect may be found in a breast cancer mass, but the pathological significance of the nonenhanced area is not entirely clear.

Other reasons for a positive margin include technical problems, but in this study, there were no findings that raised the suspicion of tumor invasion even after reviewing the final pathology specimens; therefore, we considered this to be of minor impact. Operation procedures included variations depending on each operator; however, the extent of resection and the surgical technique were performed based on specific protocols indicated in the materials and methods within this study. As for issues of the body position, it is important to have the skills to properly reflect the MRI, which is taken in the prone position, in the range of resection. Similarly, mammography is performed in a standing position with breast compression, which differs from the positioning during surgery. Therefore, caution is required in interpretation. It should also be noted that CEUS has the advantage of being able to be performed simultaneously with B‐mode US in the surgical position.

There are several limitations in this study. First, this was a small observational study. Therefore, if in situ lesions and invasive lesions were to be compared, there would be insufficient statistical power. This study evaluated the surgical margin in a cohort including both types of lesions, but some bias is inevitable. Second, it is difficult to obtain a perfect match between the imaging plane of each modality. CEUS and B‐mode US comparisons were performed with both probes fixed at the same site, allowing the evaluation of the potentially same imaging plane, whereas the comparison between MRI and B‐mode US cannot exclude the possibility that positional information differs from imaging derived from other imaging planes. Third, although the difficulty of matching imaging planes also exerts an impact, pathological findings that are not contrasted by CEUS have not been clarified because the situation may be completely different when the slice is changed for pathological verification. Therefore, further research is needed to further solidify the rationale for why a noncontrast pattern would result in positive margins.

## 5 Conclusion

In conclusion, when there is no CEUS enhancement outside the margins of the visible tumor on B‐mode, this might be a risk factor for positive surgical margins. While considering the advantages of CEUS, we emphasize the importance of integrating all imaging information, including mammography, B‐mode US, and MRI, in a comprehensive evaluation. Further detailed investigation for these issues is warranted.

## Author Contributions

**Hiroaki Shima:** conceptualization, methodology, visualization, writing – original draft, writing – review and editing. **Fukino Satomi:** data curation, investigation. **Daisuke Kyuno:** supervision. **Noriko Nishikawa:** formal analysis, software. **Satoko Uno:** formal analysis, software. **Yuta Kondo:** data curation, investigation. **Ai Noda:** data curation, investigation. **Takashi Nakamura:** investigation, visualization. **Toru Mizuguchi:** supervision.

## Ethics Statement

This study complies with the guidelines of the Surgery Journal Editors Group. This study focuses on breast cancer, and since male breast cancer is extremely rare, all cases included in the study are female. This is stated in the main text. This study adhered to ethical tenets of The Declaration of Helsinki and Ethical Principles for Medical Research Involving Human Subjects, was approved by the Clinical Trial Center of Sapporo Medical University, Japan, and is registered with UMIN‐CTR (UMIN000053287). The consent documents approved by the review committee were given to the subjects (352‐115), and the need for informed consent was waived in view of the retrospective and observational nature of the study. This study adhered to ethical tenets of The Declaration of Helsinki and Ethical Principles for Medical Research Involving Human Subjects, was approved by the Clinical Trial Center of Sapporo Medical University, Japan, and is registered with UMIN‐CTR (UMIN000053287). The consent documents approved by the review committee were given to the subjects (352‐115), and the need for informed consent was waived in view of the retrospective and observational nature of the study.

## Consent

An opt‐out consent process was used, and disclosures were made on the University's website (https://web.sapmed.ac.jp/byoin/rinshokenkyu/koukai/). Currently, the opt‐out period has ended, and it has been removed from this website. The above‐mentioned matters are also covered in relation to publishing.

## Conflicts of Interest

The authors declare no conflicts of interest.

## Data Availability

The data that support the findings of this study are available from the corresponding author upon reasonable request.
